# COVID-19 Vaccine Hesitancy among Economically Marginalized Hispanic Parents of Children under Five Years in the United States

**DOI:** 10.3390/vaccines11030599

**Published:** 2023-03-06

**Authors:** Celia Fisher, Elise Bragard, Purnima Madhivanan

**Affiliations:** 1 Department of Psychology, Fordham University, Bronx, New York, NY 10458, USA; 2 Department of Health Promotion Sciences, Mel and Enid Zuckerman College of Public Health, University of Arizona, Tucson, AZ 85721, USA

**Keywords:** COVID-19, vaccine hesitancy, Latino, pediatric, low income, health disparities, acculturation

## Abstract

Hispanic children in the US have high rates of COVID-19-related hospitalizations and deaths. Following FDA emergency approval, COVID-19 vaccination rates for young children under five years have been alarmingly low, especially in border states with significant Hispanic populations. This study identified social and cultural determinants of COVID-19 vaccine hesitancy among economically marginalized Hispanic parents of children under five. In 2022, following FDA approval, 309 Hispanic female guardians in US border states responded to an online survey assessing parental intent to vaccinate their child, demographic characteristics, COVID-19 health and vaccine beliefs, trust in traditional sources of health information, physician and community support, and acculturation to Anglo American norms. The majority (45.6%) did not intend to vaccinate their child or were unsure (22.0%). Kendall’s *tau-b* indicated vaccine acceptance was negatively associated with COVID-19 specific and general vaccine distrust, belief the vaccine was unnecessary, time living in the U.S., and language acculturation (range *t_b_* = −0.13 to −0.44; *p* = 0.05–0.001) and positively related to trust in traditional resources, doctor’s recommendation, child’s age, household income and parent education (range *t_b_* = 0.11 to 0.37; *p* = 0.05–0.001). This research highlights the importance of COVID-19 vaccination public health strategies that draw on Hispanic cultural values, community partnerships and enhanced pediatrician communication regarding routine and COVID-19-specific vaccinations.

## 1. Introduction

On 17 June 2022, the U.S. Food and Drug Administration (FDA) authorized the Pfizer-BioNtech and Moderna COVID-19 (SARS-CoV-2) vaccines for emergency use in children six months–four years of age [1]. As of February 16, 2023, there have been over 15.4 million cases of COVID-19 among children under five [2]. During the 2022 sub-variant wave, children under five had a higher COVID-19-associated hospitalization rate than other age groups and approximately half who were hospitalized had no underlying condition [3,4,5]. Moreover, infants and children under 5 are more likely to experience fatigue and loss of taste and smell for more than 4 weeks following infection [3,4,5]. Since the beginning of the pandemic, Hispanic children <18 years of age have had higher rates of hospitalization and related deaths than non-Hispanic white children [6,7], and 27% of Hispanic children under five years who were hospitalized for SARS-CoV-2 had multisystem inflammatory syndrome [4,8].

The percentage of parents who will vaccinate their younger children is a question of public health concern. Since the vaccine was approved for children five–eleven years old in October 2021, US vaccination rates for this age group have been at only 36.8% for the first dose and 30% for series completion [3,4]. These percentages are somewhat lower than national studies conducted in the months prior to FDA approval, indicating approximately 40% of parents intended to vaccinate their 5–11 year-old child against COVID-19 [9,10,11]. Later studies suggested the percentages would be similar or lower for children under the age of five [11,12]. However, it appears that estimates based on parental intent may be overly optimistic. Since FDA approval, COVID-19 vaccination rates for children under five have been lower than prior estimates [13]. As of 3 August 2022, only 5% of children six months–four years had received at least one dose of the COVID-19 vaccine, and numbers nationwide have been decreasing at an alarming rate [2]. Although vaccination rates for this age group vary by state, to date they are particularly low in border states such as Texas (1.1%), Arizona (2.1%) and New Mexico (2.4%), where persons of Hispanic heritage make up a significant portion of the population [14]. Moreover, Hispanic families are increasingly moving to rural areas of the US, where they experience higher COVID-19-related death rates and lower vaccination rates [15,16].

Little is known about how parental attitudes and beliefs have and will continue to affect COVID-19 vaccination rates for younger Hispanic children. In national studies that included samples of Hispanic respondents, application of the health beliefs model (HBM) and related theories of planned health behaviors [17,18,19,20] indicate that the most consistent predictors of parental intentions for COVID-19 vaccine uptake for children were misinformation, underestimation of disease severity and susceptibility, distrust of vaccine safety and efficacy, a lack of community support for vaccinating children against infection [9,10,11,12], lower income and education, and lack of private insurance [17,18,21,22]. These factors have also been identified among Hispanic parents as predictive of routine and influenza pediatric vaccination intent [21,23,24,25,26,27].

To date, sociocultural factors influencing Hispanic parents’ attitudes toward COVID-19 pediatric vaccinations for young children have been unexplored. The culture-centered approach argues that in addition to health beliefs and structural factors, health behaviors must be understood within the context of cultural beliefs and values [28,29,30,31]. For example, features of Hispanic culture such as *familism* and religiousness may serve to buffer against health disparities associated with structural factors such as economic and educational marginalization [32,33]. However, the positive effects of culture on health may be reduced with increased years living in the US. Acculturation describes the cultural adaptation of individuals of Hispanic heritage living in the U.S. to perceived mainstream Anglo-American activities and values [28,34]. In addition to nativity and years living in the US, measures of acculturation include language use and preferences and a psychological attachment to and belonging within the Anglo-American and Hispanic cultures [35,36]. Years living in the US and acculturation have been associated with lower odds of influenza vaccination, greater frequency of smoking, unhealthy diet, less physical activity, hypertension, and obesity among Mexican-identified and other Hispanic/Latino adults [28,37,38,39,40,41,42], and acculturation may have similar effects on parental COVID-19 vaccine hesitancy.

There are limited data on the intentions of Hispanic parents to vaccinate their children under five years against COVID-19 infection. The aim of the present study was to assess the extent to which previously identified factors found to predict parental COVID-19 vaccination decisions for children and acculturation factors associated with health behaviors among Hispanic adults are related to the intention of economically marginalized Hispanic parents living in US border states to plan to vaccinate their children under five against COVID-19.

## 2. Materials and Methods

### 2.1. Study Setting

In July 2022, following the June 17 FDA emergency approval of the COVID-19 vaccine for children six months to four years of age, online survey data were collected for 309 Hispanic female guardians (≥21 years old) of children one–four years of age. Inclusion criteria included living in the U.S. for >five years (to ensure parents had the opportunity to experience healthcare prior to and during the pandemic), household income under $55,000, and residence in the border states Arizona, Texas, and New Mexico. Participants could complete the survey in English or Spanish: 1.6% chose the Spanish option. The age range of one–four years was selected since younger infants would be experiencing regularly scheduled routine pediatric vaccination regimens that might increasingly include COVID-19 [43,44].

### 2.2. Study Instrument

**Figure 1:**
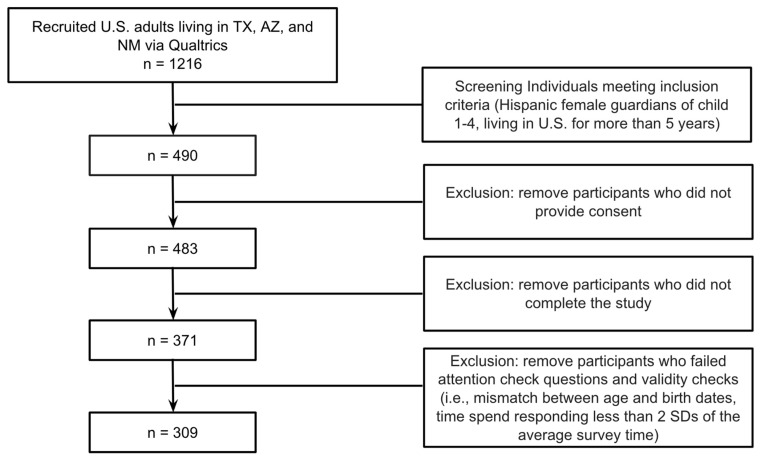
Flow diagram showing the recruitment process.

Recruitment and data collection were conducted through Qualtrics XM, a survey aggregator that recruits individuals who sign up to take paid surveys. Individuals who clicked on a link describing a survey related to children’s health viewed a screener, and those who qualified were able to access an informed consent page. The screener and consent page described the goal of the study as understanding parental attitudes toward vaccinating their children against COVID-19 infection. Individuals who consented were then sent to the survey link. Of the 1216 who responded to the screener, 490 met inclusion criteria, 7 did not provide consent, 112 did not complete the survey, and 62 failed attention and validity checks. The final sample size of 309 was sufficient to detect a medium to large effect size based on the recommended 10–20 participants per factor for logistic regression [45]. The protocol was reviewed by representatives of a Hispanic health advocacy group and approved by the Fordham University institutional review board (see Figure 1).

### 2.3. Study Variables

Demographic items included parents’ racial and ethnic background, household income, years living in the US, parent’s education, country of birth, perceived financial security, employment status, state and geographic region (rural, suburban, urban), the age and gender of the target child (their “youngest child between the ages of one–four”), which routine pediatric vaccinations their child had received [43,44], and whether the target child received government nutritional assistance (SNAP) and had health insurance.

The primary outcome measure was parental intention to vaccinate their one–four-year-old child against COVID-19 infection. This single item began with “The FDA has approved the COVID-19 vaccine for children 6 months and older. The vaccine requires children to get at least 2 shots.”. Response options included whether the parent would “definitely not,” “probably not,” was “unsure”, or would “probably” or “definitely” vaccinate their child [9,12,41]. For Chi Square and multinomial logistic regression analyses, the item was recoded into three categories: 1 = resistant (definitely or probably not), 2 = unsure, and 3 = accepting (probably or definitely will).

Perceived susceptibility to COVID-19 infection was measured with a question asking parents how likely it was that their child would be infected with COVID-19 in the future, measured on a 4-point scale (1 = not at all likely, 4 = very likely). To assess perceived infection severity, parents were asked whether their child had ever had COVID-19 and then reported the actual or anticipated severity of the child’s symptoms through three options: 1 = Mild (e.g., cough, fever, body aches), 2 = More serious (e.g., difficulty breathing) that got better with medicine, and 3 = Very serious (e.g., hospitalization, longer term effects). The actual/anticipated responses were combined into one “perceived severity” variable.

Five items adapted from existing scales assessed knowledge and misconceptions about the COVID-19 vaccine on a 6-point Likert-type scale (1 = strongly disagree–6 = strongly agree) [21,46,47]. Items included beliefs that getting the vaccine would protect the child, protect family and community, cause the infection, cause long-term health problems, and that getting the infection was a better source of immunity. Scale scores were calculated as the mean of all items, with knowledge items reverse scored (inter-item reliability *α* = 0.76). A second six-point Likert-type four-item scale assessed parents’ beliefs that the COVID-19 vaccine was no longer necessary because: herd immunity has been reached; the vaccine no longer works because vaccinated adults have gotten COVID-19; children are naturally immune to the virus; and most people no longer get COVID-19 (reliability: *α* = 0.79) [12]. A nine-item General Vaccine Distrust Scale [9,48] was scored on a six-point Likert scale (1 = strongly disagree–6 = strongly agree). Sample items included “Drug companies cover up the dangers of vaccines recommended for young children,” “People are deceived about whether vaccines actually work for young children.” The scale score based on the average of the item means yielded an inter-item reliability of *α* = 0.89.

Parents’ trust in traditional resources, including national health agencies (FDA and CDC), their local health department, news, social media, and their doctor or pharmacist, was assessed on a six-point scale (1 = strongly disagree–6 = strongly agree; *α* = 0.80). To assess physician COVID-19 recommendations, parents selected among three options: child’s doctor had recommended their child get the COVID vaccine, did not mention the vaccine, or recommended against vaccination. For Chi-square and multinomial logistic regression, the item was coded as 1 = Doctor did not mention or recommended against the vaccine and 2 = Doctor recommended vaccination. The five-item Community Support Scale asked parents to rate how supportive their religious and political leaders, physicians, other parents and family members were for vaccinating children between the ages of one and four years against COVID-19 (0 = strongly unsupportive–5 = strongly supportive) [48,49]. A scale score was calculated as the mean of the six items (*α* = 0.71).

In addition to nativity and time living in the US, the survey included three measures of acculturation. The six-item Short Acculturation Scale for Hispanics (SASH) [35,50] assesses language preferences (1 = Spanish only to 5 = English only) across different contexts: at home, with friends, TV/film/radio/online programs, news, usual conversations and preferred conversations with their doctor. Four items from the Psychological Acculturation Scale (PAS) [36] assessed whether participants felt more of a sense of belonging with 1 = Hispanic/Latino culture, 2 = both Hispanic/Latino culture and Anglo-American culture, or 3 = Anglo-American culture. An example of one of the items was “In your opinion‚ which group of people best understands your ideas (your way of thinking)?” Scale scores were calculated as the mean of all items (SASH, *α* = 0.90; PAS, *α* = 0.76). Since persons living in rural areas may rely on nearby pharmacies rather than distant clinics, we administered a doctors/healthcare workers and a pharmacist version of the five-item Group-Based Medical Mistrust Scale [51] scored on a six-point Likert-type scale (1 = strongly disagree–6 = strongly agree). Examples of the items include “Hispanic/Latino people cannot trust doctors and health care workers/pharmacists”, “I have personally been treated poorly or unfairly by doctors or healthcare workers/pharmacists because I am Hispanic Latino.” Responses from both scales were combined to create a 10-item scale (*α* = 0.77).

### 2.4. Data Analysis Plan

Descriptive statistics were calculated, followed by Chi square tests and Analysis of Variance to determine differences among *accepting*, *unsure*, and *resistant* parents based on demographics, health beliefs, and structural and acculturation measures using IBM SPSS version 28.0.10 (142) © IBM Corporation, 2021. Kendall’s *tau-b* correlations examined associations among the 3 vaccine intention groups, health beliefs, structural, cultural, and demographic factors, followed by a multinomial logistic regression analysis to determine independent effects of correlated variables with vaccination intent (adjusted odds ratios at 95% confidence intervals).

## 3. Results

### 3.1. Demographic Data

Means, standard deviations and percentages along with Chi square and ANOVA significance values for the demographic data from the total sample and intent-to-vaccinate categories are presented in Table 1. The mean age of parents was 29.55 years (*SD* = 6.71; range = 21–60), with accepting parents significantly older. Close to a third of respondents reported income under USD 20,000. Based on reported household size, 141 (45.6%) households met US poverty thresholds [52]. Significantly more accepting parents reported a college education, higher household income, and financial security and were more likely to be employed than other groups. Approximately a quarter of the parents lived in rural areas, and a third lived in suburban and in urban areas. Most participants (66.0%) resided in Texas, followed by Arizona (25.6%), and New Mexico (8.4%). Most parents (68.6%) were born in the US and were of Mexican ethnicity (61.2%). The children’s age was equally distributed across one, two, three, and four years (*M* = 32.72 months, *SD* = 13.81). Most children had either government or private insurance, and half the children received nutritional support from SNAP. Less than half of the parents indicated their child had received all seven recommended routine vaccinations; however, between 9.4 and 21.7% indicated they did not know if their child had been vaccinated. Only a third indicated they probably or definitely would vaccinate their child annually if the COVID-19 shot became a seasonal vaccine.

### 3.2. Intent to Vaccinate Child against COVID-19

Of the 309 participants, 141 (45.6%) would definitely or probably not vaccinate their child (resistant parents), 68 (22.0%) were unsure parents, and 100 (32.4%) would probably or definitely vaccinate their child against COVID-19 (accepting parents).

### 3.3. Descriptive Statistics

**Figure 2:**
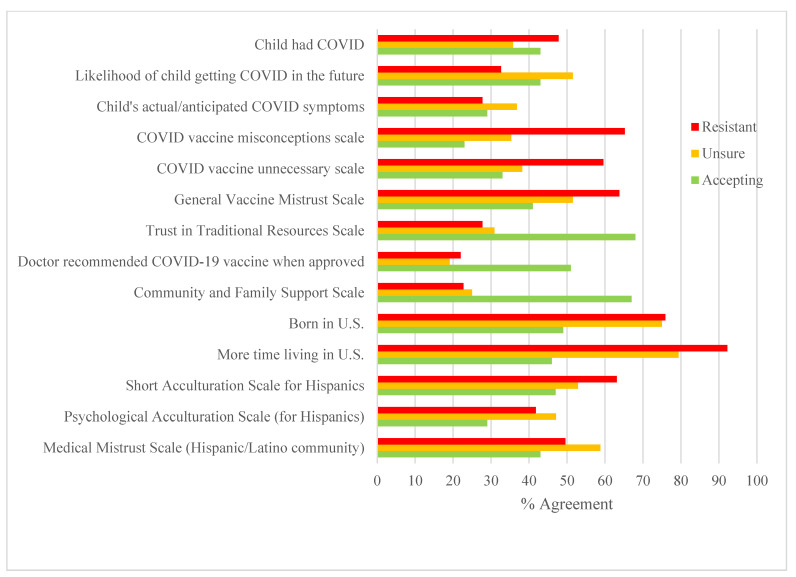
Percent of resistant, unsure, and accepting parents reporting agreement with health beliefs, physician and community support, cultural factors and medical mistrust. Note: For Figure 2, continuous scales were recoded into dichotomous variables using median split, with the percentages in the graph reflecting agreement (scores above the median). Likelihood of getting COVID-19 illustrates the percentage of parents reporting that it was likely or very likely that their child would contract COVID-19. Child’s actual/anticipated COVID-19 symptoms illustrates the percent of parents reporting their child had/would have moderate or severe COVID-19 symptoms. More time living in the U.S. illustrates parents who had been in the US 5 years or more.

Descriptive statistics for health beliefs, structural factors and acculturation measures for the total sample and by the three COVID-19 intent categories are provided in Table 2 and Figure 2. A MANOVA with the three intent categories as the independent variable and continuous social determinant variables as dependent variables was significant, F(22,594) = 8.98, *p* < 0.001, ETS = 0.250), followed by bivariate analyses (see Table 2) and Scheffé tests (*p* < 0.05). Resistant compared to unsure and accepting parents were significantly less likely to believe their child was susceptible to COVID-19, more likely to hold COVID-19 vaccine misconceptions, believe the vaccine was unnecessary, hold a general mistrust of vaccines, and to prefer to speak English. In comparison to both resistant and unsure parents, accepting parents had higher scores on the scales that measured trust in traditional health resources, had greater community support, were more likely to live in urban compared to rural settings, have been born outside the US and to have lived in the US for a shorter period of time. Resistant parents were almost twice as likely to indicate that they felt a lack of support for the pediatric COVID-19 vaccine from their child’s doctor (21.3%) compared to unsure (8.3%) and accepting parents (7.0%), (*X*^2^(2) =10.88, *p* < 0.01). Accepting parents were more than twice as likely (51.0%) compared to unsure (19.1%) and resistant (22.0%) parents to report that their doctor had recommended the COVID-19 vaccine.

### 3.4. Correlations and Multinomial Logistic Regression

Kendall’s *tau-b* correlations among the three-level COVID-19 vaccine intent categories (resistant, unsure, accepting) and demographic, attitudinal, structural and cultural factors are provided in Table 3. Vaccine acceptance was significantly and positively associated with perceived child COVID-19 susceptibility, trust in traditional health resources, a doctor’s recommendation, community support, child’s age, financial security, higher parental income and education, and if the child had private compared to government or no health insurance. Vaccine acceptance was significantly and negatively associated with vaccine misconceptions and belief that the vaccine was unnecessary, general vaccine mistrust, time living in the US, nativity, and higher scores on language acculturation.

As illustrated in Table 4, a multinomial logistic regression with accepting parents as the reference group was conducted to examine factors significantly associated with parental intent in the correlational analyses described above. The factors explained 61.3% of the variance (Nagelkerke *R*^2^) in plans to vaccinate one’s child against COVID-19. Parents were more likely to be in the resistant group than the accepting group if they had more COVID-19 vaccine misconceptions, lower trust in traditional resources, less community support, longer time living in the U.S, and lower education. Parents were more likely to be in the unsure group than the accepting group if they had not received a doctor’s recommendation, reported lower community support and household income, and had spent longer time living in the U.S.

## 4. Discussion

Since the beginning of the COVID-19 pandemic, Hispanic children have had higher rates of hospitalization and deaths than non-Hispanic white children. These statistics underscore the public health urgency of increasing pediatric vaccine acceptance for this population. In the month following FDA emergency approval of the COVID-19 vaccine for children under five years of age, the majority of economically marginalized Hispanic female guardians who responded to the survey did not intend to or were unsure if they would vaccinate their young children against COVID-19 infection. These percentages are consistent with a recent study of parents of one–four year old children, including Hispanic and other ethnic groups, conducted just prior to FDA approval [12]. The low rate of parental vaccine acceptance is especially concerning given the low initial turnout since approval, especially in US border states where this survey was conducted [14]

Demographic factors identified in the literature were significantly associated with parents’ vaccination intentions. For example, the child’s age was positively associated with intention to vaccinate one’s child. This is consistent with results from a prior qualitative study in which the narratives of an ethnically diverse group of parents of children one to four years of age indicated that infants and toddlers were perceived to be more vulnerable to side-effects from newly tested vaccines, and in some cases, less susceptible to infection because parents could more easily restrict their environments [12]. Despite the relatively restricted household income range selected for this study, income level and financial security along with educational attainment were positively associated with intention to vaccinate. This is due in part to the fact that within this lower income sample, there was significant variation, with nearly half the respondents meeting the US government poverty threshold and reporting a high school or lower level of education. Findings that indicated that higher percentages of resistant and unsure parents lived in rural communities as compared to accepting parents are consistent with prior research demonstrating that rural communities have lower vaccination rates for preventable diseases, and this has magnified during the pandemic [15,16,53].

Consistent with prior studies on parental health beliefs, resistant parents were more likely than the unsure and accepting parents to hold COVID-19 vaccine misconceptions, believe their child was not susceptible to infection, that COVID-19 symptoms would be less severe, that the vaccine would be harmful to their child and unnecessary, and hold higher levels of general vaccine mistrust [9,12,23,24,25,54]. COVID-19 vaccine misinformation, conspiracy theories and hoaxes have been pervasive in the general mass media as well as in social media posts specifically targeting Hispanic communities [55,56,57]. Hispanic female guardians may be particularly susceptible to misinformation presented in these online narratives because of a documented history of coerced sterilization in the U.S., especially among those of Mexican descent [55,56,58]. Resistant parents were also more likely to believe the vaccine was not necessary to protect their children. These attitudes may reflect recent media reports that the Omicron variant symptoms are less severe than the earlier variants, that children are at lower risk than adults, and that infection and reinfection are now more common among vaccinated adults [12].

Nativity, years in the US and acculturation have been identified as risk factors for influenza vaccination, cancer and cardiac health among Hispanic adults [39,40,53]. This is the first study to document these factors as barriers to parental intention to vaccinate their young children against COVID-19. Across the sample, resistant parents were significantly more likely to have been born in and lived longer in the US and to endorse English versus bilingual or Spanish preferences compared to either accepting or unsure parents. Although more resistant parents reported a sense of belonging to Anglo culture, overall, few parents selected Anglo-only culture in response to items on the cultural belonging scale. This suggests that this population maintains a strong sense of belongingness with the Hispanic culture, consistent with research indicating that although certain features of Hispanic culture may become more acculturated, aspects such as *familism* and religiosity remain important [59]. Relatedly, perceived discrimination of Hispanic patients by healthcare providers was low in this sample and not significantly associated with vaccination intent, suggesting that Hispanic cultural constructs such as *confianza* (trust) and *respeto* (deference to) toward the authoritative position of health care providers [60,61] may buffer the negative effect of acculturation on pediatric vaccine intentions.

The data also underscore the importance of community support in pediatric vaccination decisions [62], with accepting parents indicating greater community support. Targeted approaches for Hispanic border state parents may thus benefit from messaging developed in partnership with and delivered through existing trusted community coalitions, such as the Hispanic Federation VIDA Initiative [63] and other Hispanic/Latino grassroot initiatives.

This study adds to existing research by highlighting the role that attitudes toward healthcare providers may play in pediatric COVID-19 hesitancy. Compared to accepting and unsure parents, resistant parents were almost twice as likely to respond that they felt a lack of support from their child’s doctor and that they would not rely on their doctor’s or pharmacist’s recommendation for vaccinating their child against COVID-19. Overall, 49.5% of the parents indicated that their pediatrician had never mentioned the COVID-19 vaccine for their child, and resistant parents were less likely than other parent groups to indicate that their doctor had recommended their child be vaccinated against COVID-19 infection. Although national statistics indicate that approximately 90% of US Hispanic children receive at least the first dose of recommended routine vaccinations during the first 24 months of life [64,65], in the current study, less than half of the parents indicated that their child had received all the recommended vaccinations, and many indicated that they did not know if their child had received the vaccination. These responses underscore the importance of pediatricians serving Hispanic families to take a more active role in discussing with parents both routine infant and childhood vaccinations and the COVID-19 vaccine specifically [23,62].

This is the first study to explicitly focus on pediatric COVID-19 vaccine hesitancy among economically marginalized Hispanic parents of children under the age of five. Its strengths lie in the examination of the influence of health beliefs, structural factors, and acculturation on parents’ intentions. The study is not without limitations. We selected female guardians because data show they are more likely than male guardians to make the majority of healthcare decisions for their children [66,67]. However, these decisions, especially within the context of the cultural construct of *familism,* may include important male guardian perspectives. Similarly, we sampled parents from three border states with large Hispanic, predominantly Mexican, populations. Thus, these findings may not be generalizable to lower income Hispanic parents in other regions and of other cultural heritages. Although the sample was diverse with respect to nativity, immigration history, and language preference, less than two percent chose to take the survey in English. This may be a function of the use of the Qualtrics system, as individuals who may have signed up for a variety of surveys may be more fluent in English.

## 5. Conclusions

This is the first study to apply the culture-centered model to understand pediatric COVID-19 vaccination hesitancy for economically marginalized Hispanic children under five years of age. The risk of acculturation to pediatric COVID-19 vaccination may be especially potent for Hispanic adults living in Texas, Arizona, and New Mexico—states with a relatively low vaccine uptake and more conservative political views towards vaccinations. Lack of community support, loss of faith in government and doctors as trusted healthcare resources, and inadequate public health messaging regarding the value of vaccination, despite the increase in infection among formerly vaccinated adults, may further exacerbate these misconceptions. The data suggest that public health strategies that draw on Hispanic community partnerships and on early and enhanced pediatrician-parent communication about routine and COVID-19-specific vaccinations are important public health strategies for successfully fighting infection during the COVID-19 pandemic and future pandemics.

## Figures and Tables

**Table 1:** Frequencies/percentages and means/standard deviations for parental demographic characteristics and non-parametric analyses across COVID-19 vaccine intent subgroups.

		COVID-19 Vaccine Intention			
	Total Sample *N* = 309	Resistant *n* = 141 (45.6%)	Unsure *n* = 68 (22.0%)	Accepting *n* = 100 (32.4%)	
	*n(%)*	*n(%)*	*n(%)*	*n(%)*	
Parent age, *M (SD),* Range = 21–60-years	29.55 (6.71)	28.30 (6.76)	28.87 (5.55)	31.77 (6.85)	*F*(2,306) = 8.68 ***
Child’s age (youngest 1–4-years)					*X*^2^ =15.68 *
1-year	77 (24.9)	45 (31.9)	18 (26.5)	14 (14.0)	
2-years	99 (32.0)	44 (31.2)	24 (35.3)	31 (31.0)	
3-years	80 (25.9)	36 (25.5)	14 (20.6)	30 (30.0)	
4-years	53 (17.2)	16 (11.3)	12 (17.6)	25 (25.0)	
Education					*X*^2^ = 34.90 ***
No college	139 (45.0)	85 (60.3)	32 (47.1)	22 (22.0)	
Some college or higher	170 (55.0)	56 (39.7)	36 (52.9)	78 (78.0)	
Annual household income					*X*^2^ = 22.69 ***
<$20,000	84 (27.2)	44 (31.2)	29 (42.6)	11 (11.0)	
$20,000-$29,999	106 (34.3)	45 (31.9)	18 (26.5)	43 (43.0)	
$30,000-$54,999	119 (38.5)	52 (36.9)	21 (30.9)	46 (46.0)	
Financial security					*X*^2^ = 11.28 *
Cannot make ends meet	67 (21.7)	39 (27.7)	14 (20.6)	14 (14.0)	
Have just enough	161 (52.1)	71 (50.4)	40 (58.8)	50 (50.0)	
Comfortable	81 (26.2)	31 (22.0)	14 (20.6)	36 (36.0)	
Child’s health insurance					*X*^2^ = 35.42 ***
No insurance	22 (7.1)	6 (4.3)	5 (7.4)	11 (11.0)	
Government insurance	200 (64.7)	113 (80.1)	44 (64.7)	43 (43.0)	
ACA/private insurance	87 (28.2)	22 (15.6)	19 (27.9)	46 (46.0)	
Child nutrition benefits (SNAP)					
Yes	165 (53.4%)	83 (58.9%)	37 (54.4%)	45 (45.0%)	
Neighborhood					*X*^2^ = 11.29 *
Rural	72 (23.3)	38 (27.0)	18 (26.5)	16 (16.0)	
Suburban	118 (38.2)	60 (42.6)	25 (36.8)	33 (33.0)	
Urban	119 (38.5)	43 (30.5)	25 (36.8)	51 (51.0)	
Region					*X*^2^ = 16.78 **
Texas	204 (66.0)	104 (73.8)	48 (70.6)	52 (52.0)	
Arizona	79 (25.6)	32 (22.7)	13 (19.1)	34 (34.0)	
New Mexico	26 (8.4)	5 (3.5)	7 (10.3)	14 (14.0)	
Employment Status					*X*^2^ = 9.86 **
Not employed	109 (35.3)	57 (40.4)	29 (42.6)	23 (23.0)	
Employed part-time/full-time	200 (64.7)	84 (59.6)	39 (57.4)	77 (77.0)	
Intent to Provide COVID-19 Seasonal Vaccine					*X*^2^ = 106.23 ***
Agree	96 (31.1%)	8 (5.7%)	20 (29.4%)	68 (68.0%)	

*Note*. * p < 0.05; ** *p* < 0.01; *** *p* < 0.001.

**Table 2:** Means/standard deviations and frequencies/percentages for social determinants and analyses of variance across COVID-19 vaccine intent subgroups.

	COVID-19 Vaccine Intentions				
	Total Sample *N* = 309	Resistant *n* = 141 (45.6%)	Unsure *n* = 68 (22.0%)	Accepting *n* = 100 (32.4%)	*F*
	*M (SD)*	*M (SD)*	*M (SD)*	*M (SD)*	
Perceived Susceptibility					
Child had COVID, *n (%)*	131 (43.5)	64 (47.8)	24 (35.8)	43 (43.0)	*X^2^* = 2.61 *n.s.*
Likelihood future COVID infection (child)	2.28 (0.88)	2.13 (0.86)	2.53 (0.84)	2.33 (0.89)	5.19 **
Perceived Severity					
Child’s actual/anticipated COVID symptoms	1.37 (0.61)	1.35 (0.61)	1.46 (0.66)	1.34 (0.57)	0.90 *n.s.*
Vaccine Misconceptions					
COVID vaccine misconceptions	3.46 (1.14)	4.11 (0.95)	3.32 (0.74)	2.64 (1.05)	72.69 ***
COVID vaccine unnecessary	3.21 (1.17)	3.61 (1.05)	3.06 (0.94)	2.76 (1.28)	18.23 ***
Trust					
General Vaccine Mistrust	3.21 (1.06)	3.52 (1.02)	3.17 (0.92)	2.82 (1.08)	13.80 ***
Trust in Traditional Resources	3.86 (1.02)	3.43 (1.05)	3.82 (0.71)	4.49 (0.81)	39.94 ***
Structural Influences					
Doctor recommended COVID-19 vaccine, *n (%)*	95 (30.7)	31 (22.0)	13 (19.1)	51 (51.0)	*X^2^* = 28.67 ***
Community/Family Support Scale	2.88 (0.60)	2.66 (0.62)	2.81 (0.48)	3.25 (0.45)	35.27 ***
Acculturation					
Born in U.S.	207 (67%)	107 (75.9%)	51 (75.0%)	49 (49.0%)	*X^2^* = 21.65 ***
Time Living in U.S. ^a^	3.32 (1.03)	3.72 (0.71)	3.46 (0.95)	2.66 (1.13)	39.71 ***
SASH	3.70 (0.87)	3.84 (0.86)	3.66 (0.89)	3.53 (0.83)	3.93 *
PAS	1.64 (0.49)	1.67 (0.53)	1.67 (0.49)	1.58 (0.44)	1.12 *n.s.*
Medical Mistrust Scale	3.11 (0.79)	2.09 (0.85)	3.23 (0.68)	3.07 (0.76)	0.96 *n.s.*

Note. * p < 0.05; ** *p* < 0.01; *** *p* < 0.001. ^a^ Time in US.

**Table 3:** Kendall’s *tau-b* correlations among the three levels of COVID-19 vaccine intent, health beliefs, demographic, structural and cultural factors.

	1	2	3	4	5	6	7	8	9	10	11	12	13	14	15	16	17	18
1. COVID-19 vaccine intent (3-level)	--																	
2. Child’s COVID-19 history (*n* = 301)	−0.05	--																
3. Likelihood of future COVID-19 infection (child)	0.11 *	0.27 ***	--															
4. Severity of child’s COVID-19 symptoms (past or hypothetical)	0.02	0.02	0.21 ***	--														
5. COVID-19 Vaccine Distrust Scale	−0.44 ***	0.04	0.01	−0.03	--													
6. COVID-19 Vaccine Unnecessary Scale	−0.26 ***	−0.00	0.02	−0.01	0.47 ***	--												
7. General Vaccine Distrust Scale	−0.21 ***	−0.05	0.03	0.08	0.40 ***	0.49 ***	--											
8. Trust in Traditional Resources Scale	0.37 ***	−0.00	0.09 *	0.03	−0.25 ***	−0.01	−0.08 ***	--										
9. Doctor recommended COVID-19 vaccine	0.24 ***	−0.04	0.06	0.01	−0.19 ***	−0.06	−0.03	0.24 ***	--									
10. Community Support Scale	0.35 ***	0.07	0.09	−0.03	−0.26 ***	−0.09 *	−0.07 **	0.35 ***	0.19 ***	--								
11. Time living in U.S.	−0.39 ***	−0.02	−0.15 **	−0.10	0.19 ***	−0.10 *	−0.05	−0.41 ***	−0.33 ***	−0.34 ***	--							
12. Language Acculturation Scale	−0.13 *	0.00	−0.06	−0.10 *	0.05	−0.07	−0.10	−0.17 ***	−0.16 **	−0.07	0.32 ***	--						
13. Cultural Belonging Scale	−0.07	−0.06	0.05	0.01	0.01	−0.01	−0.04	−0.05	0.12 *	−0.06	0.08 **	0.16 ***	--					
14. Medical Mistrust Scale	−0.01	−0.01	0.05	0.10	0.05	0.12 *	0.18 ***	−0.01	−0.01	−0.00	−0.12 *	−0.13 **	−0.04	--				
15. Household income	0.12 *	0.19 **	0.09 *	−0.04	0.01	0.00	−0.04	0.10 *	0.05	0.02	−0.17 **	−0.04	−0.04	−0.09	--			
16. Child age in months	0.17 **	0.06	0.07	0.05	−0.12 *	−0.10 **	−0.11 ***	0.06	0.09	−0.03	−0.10	0.05	0.02	−0.07	0.15 **	--		
17. Parent education	0.34 ***	0.06	−0.02	−0.04	−0.13	0.02	−0.02	0.29 ***	0.17 **	0.26 ***	−0.40 ***	−0.16 *	−0.04 *	0.08	0.20 ***	0.09		
18. Child insured	0.11 *	−0.11	−0.04	−0.00	−0.06	−0.01	0.02	0.06	0.06	0.07	0.09	−0.16 ***	0.02	0.04	0.02	−0.01	0.08	
19. Neighborhood	0.16 **	−0.06	−0.01	0.02	−0.10 *	−0.01	−0.02	0.14 **	0.17 ***	0.20 ***	−0.23 ***	−0.10 *	0.03	−0.04	0.05	0.14 **	0.14 **	0.15 **

Note. * p< 0.05; ** *p* < 0.01; *** *p* < 0.001.

**Table 4:** Adjusted multinominal logistic regressions predicting COVID-19 vaccination intention for child aged 1 to 4 years.

	Resistant vs. Accepting *				Unsure vs. Accepting *			
Variable	OR [95% CI]	*p*-Value	aOR [95% CI]	*p*-Value	OR [95% CI]	*p*-Value	aOR [95% CI]	*p*-Value
Likelihood of future COVID-19 infection (child)	0.76 [0.56,1.03]	0.07	0.73 [0.45,1.17]	0.19	1.20 [0.91,1.86]	0.15	1.40 [0.88,2.21]	0.16
COVID-19 Vaccine Misconceptions Scale	5.42 [3.64,8.06]	<0.001	3.91 [2.10,7.28]	<0.001	2.08 [ 1.47,2.94]	<0.001	1.59 [0.88,2.89]	0.12
COVID-19 Vaccine Unnecessary Scale	1.99 [1.55,2.56]	<0.001	1.62 [0.93,2.83]	0.09	1.28 [0.97,1.70]	0.08	1.00 [0.58,1.72]	0.99
General Vaccine Distrust Scale	1.95 [ 1.49,2.55]	<0.001	0.93 [0.52,1.63]	0.80	1.38 [ 1.02,1.87]	0.03	1.05 [0.59,1.87]	0.86
Trust in Traditional Resources Scale	0.25 [ 0.17,0.36]	<0.001	0.52 [0.30,0.90]	0.02	0.38 [0.26,0.57]	<0.001	0.76 [0.44,1.31]	0.32
Doctor did not recommend COVID-19	3.69 [2.11,6.46]	<0.001	1.15 [0.47,2.80]	0.77	4.40 [2.14,9.05]	<0.001	2.29 [1.03,6.00]	0.04
Community Support Scale	0.12 [0.07,0.22]	<0.001	0.36 [0.15,0.90]	0.03	0.19 [0.10,0.36]	<0.001	0.28 [0.11,0.67]	0.004
Time living in U.S.	3.00 [2.20,4.09]	<0.001	1.88 [1.16,3.07]	0.01	1.99 [1.45,2.73]	<0.001	1.58 [1.01,2.48]	0.046
Short Acculturation Scale for Hispanics	1.53 [1.13,2.08]	0.007	0.88 [0.52,1.49]	0.63	1.17 [0.82,1.67]	0.38	0.90 [0.54,1.48]	0.67
Household income	0.73 [0.58,0.91]	0.006	0.89 [0.63,1.27]	0.53	0.61 [0.47,0.80]	<0.001	0.63 [0.44,0.89]	0.009
Child age in months	0.97 [0.95,0.99]	0.006	0.97 [0.94,1.00]	0.08	0.98 [0.95,1.00]	0.07	0.97 [0.94,1.00]	0.09
Parent education	0.41 [0.31,0.53]	<0.001	0.60 [0.39,0.91]	0.02	0.58 [0.44,0.77]	<0.001	0.95 [0.64,1.42]	0.79
No insurance	1.14 [0.37,3.49]	0.82	0.79 [0.17,3.59]	0.76	1.10 [0.34,3.60]	0.87	0.66 [0.15,2.89]	0.59
Government health insurance	5.50 [2.96,10.19]	<0.001	1.79 [0.65,4.95]	0.26	2.48 [1.26,4.89]	0.009	0.88 [0.34,2.29]	0.79
Private insurance/ACA	Ref.	Ref.	Ref.	Ref.	Ref.	Ref.	Ref.	Ref.
Suburban/Urban	0.52 [0.27,0.99]	0.047	1.28 [0.47,3.54]	0.63	0.53 [0.25,1.13]		1.04 [0.39,2.75]	0.94
Rural	Ref.	Ref.	Ref.	Ref.	Ref.	Ref.	Ref.	Ref.

Note. * Accepting is the comparison group. OR = crude odds ratio. aOR = adjusted odds ratio. 95% CI = 95% Confidence Interval. Ref. = Reference group.

## Data Availability

Data supporting the reported results are provided on the Fordham University download data portal at: https://www.fordham.edu/info/27975/human_development_and_social_justice/12628/covid-19_vaccine_hesitancy_among_economically_marginalized_hispanic_parents_of_children_under_five_years_in_the_united_states (accessed on 12 December 2022).
